# Risky online self-disclosure in adolescents: a meta-analytic review of predictors and outcomes

**DOI:** 10.3389/fpsyg.2025.1734301

**Published:** 2026-01-12

**Authors:** Jingle Sun, Akmar Hayati Ahmad Ghazali, Rahman Saiful Nujaimi Abdul

**Affiliations:** 1 Faculty of Modern Languages and Communication, Universiti Putra Malaysia, Serdang, Malaysia; 2 Faculty of Media and Communication, Mianyang Normal University, Mianyang, China

**Keywords:** adolescents, media psychology, meta-analysis, privacy calculus theory, risky self-disclosure

## Abstract

In the digital era, adolescents’ risky online self-disclosure has attracted growing scholarly attention. However, empirical findings regarding its predictors and outcomes remain inconsistent. Grounded in Privacy Calculus Theory, this study conducted a meta-analysis of 13 empirical studies (total *N* = 41,521) to systematically examine the psychological mechanisms and contextual moderators of adolescents’ risky online self-disclosure. Results showed that both benefit and cost predictors had non-significant overall effects with substantial heterogeneity, suggesting that adolescents’ risky self-disclosure is not solely driven by single psychological traits but rather shaped by multiple factors such as internet context, platform characteristics, and social interaction needs. In contrast, the overall effect of outcome variables was significant and positive, indicating a stable relationship between risky self-disclosure and its consequences. Further analyses revealed that sample size, national context, and platform type significantly moderated these relationships. This study elucidates the psychological bias mechanisms underlying adolescents’ online risk behaviors and provides practical implications for risk prevention and media literacy education in social media contexts.

**Systematic review registration:**

https://www.crd.york.ac.uk/prospero/ CRD420251117327.

## Highlights

- Conducted a meta-analysis of 13 studies (*N* = 41,521) examining adolescents’ risky online self-disclosure.
- Benefit-related and cost-related predictors showed non-significant overall effects, underscoring the complex and multifaceted nature of adolescents’ risky online self-disclosure.
- Risky self-disclosure was consistently associated with its consequences, indicating stable consequences across contexts.
- Sample size, national context, and platform type significantly moderated the relationships, revealing contextual contingencies.
- Findings provide insights into psychological mechanisms and practical implications for social media risk prevention and media literacy education.

## Research background

In recent years, adolescents’ online behaviors and social media engagement have become a major focus of research in sociology, psychology, and public policy. According to Howe (2024), statistics from 2023–2024 indicate that social media users account for approximately 83.1% of the global population, with the usage of platforms such as TikTok and Instagram increasing rapidly among younger demographics. In the digital context, social media serves not only as a space for adolescents’ daily communication and entertainment but also as a crucial arena for identity construction and self-presentation. Adolescents increasingly use multimodal symbolic resources such as text, images, and short videos to engage in display-oriented rather than narrative-oriented self-expression (Hernández-Serrano et al., 2022; Danielsen et al., 2024; Pérez-Torres, 2024).

While social media use can foster adolescents’ sense of agency, identity exploration, and peer connection (Black, 2023; West et al., 2024), their capacity for risk evaluation remains underdeveloped (Lahti et al., 2024; Kim et al., 2025). Studies suggest that adolescents often exhibit cognitive biases when assessing the potential risks of online self-disclosure, tending to overestimate its social or psychological benefits (Zhou and Liu, 2023; Kim et al., 2025). Within social media environments, self-disclosure facilitates friendship formation and reinforces identity construction (Valkenburg and Piotrowski, 2017). However, some adolescents engage in high-risk self-disclosure behaviors, such as sharing sexual content (Bobkowski et al., 2012; Eleuteri et al., 2017), posts about alcohol consumption (Scott et al., 2024), or self-harm-related content (Scherr, 2022; Choe et al., 2023; Ke et al., 2025).

Such risky self-disclosures are often motivated by a desire for social validation and peer acceptance, yet adolescents tend to neglect their potential long-term consequences, including cyberbullying, reputational damage, and the loss of educational or employment opportunities (Yang et al., 2023; Babilonová et al., 2024; Kim et al., 2025). Moreover, visual depictions of health-risk behaviors shared online can normalize and reinforce such behaviors within peer groups (Sherman et al., 2016). Overall, social media provides adolescents with valuable opportunities for self-expression and social connection; however, its anonymity, synchronicity, and content persistence can amplify the online disinhibition effect, increasing the likelihood of risky behaviors (Suler, 2004; Clark-Gordon et al., 2019). The reduction of behavioral constraints under anonymity, coupled with the long-term accessibility of online content, may further intensify the social and psychological implications of adolescents’ risky self-disclosure.

### Conceptualizing risky self-disclosure among adolescents

Self-disclosure is generally defined as the act of communicating personal information to others, including the expression of one’s attitudes, emotions, experiences, or values (Jourard and Lasakow, 1958; Cheung et al., 2015). With the widespread adoption of social media, self-disclosure has increasingly expanded into digital environments, becoming a central component of adolescents’ social interaction and identity construction. Although the concept of *self-disclosure* has been extensively examined, the definition of *risky self-disclosure* remains contested in the literature.

Taddicken (2014) distinguished between “basic information disclosure” and “sensitive information disclosure” in online contexts, with the latter involving potential risks related to privacy, reputation, or emotional harm. Similarly, Koutamanis et al. (2015) introduced the concept of *risky online self-presentation* to describe adolescents’ online behaviors that emphasize physical attractiveness or sexualized self-images, for example, posting provocative poses or revealing photos. Compared with more neutral and predictable social activities, such as sharing daily life updates or interacting with friends, such displays involve higher levels of uncertainty and social risk. Experimental evidence by Sherman et al. (2016) further indicated that uploading images depicting drinking, smoking, or inappropriate gestures can also be regarded as a form of risky self-disclosure.

From a theoretical standpoint, this study draws on Privacy Calculus Theory (Dinev and Hart, 2006) to conceptualize and explain adolescents’ risky self-disclosure. According to this theory, individuals engage in a psychological evaluation of potential risks and benefits before disclosing personal information. When the perceived benefits, such as social connection, identity expression, or peer approval, are considered to outweigh the perceived risks, including privacy loss or reputational harm, individuals are more likely to disclose. For adolescents, however, their cognitive development and risk assessment capacities are not yet fully mature, which leads to systematic biases in this risk–benefit calculation. As a result, they tend to overestimate the social rewards of self-disclosure while underestimating its potential risks (Zhou and Liu, 2023; Kim et al., 2025; Pazdur et al., 2025).

Within this framework, risky self-disclosure can be understood as a behavioral manifestation of imbalance in adolescents’ privacy calculus. Motivated by the pursuit of social validation and self-expression, adolescents may choose to share information or content that involves potential privacy, reputational, or psychological risks. The “riskiness” of such disclosure does not stem solely from its outcomes but rather from the inherent risk and cognitive bias embedded in the act itself.

Accordingly, this study defines adolescents’ risky self-disclosure as the intentional or unintentional sharing of sensitive, private, or potentially harmful personal information or content on online platforms, based on adolescents’ subjective evaluation of risks and benefits. This behavior reflects their cognitive biases and motivational conflicts within the privacy calculus, specifically the tendency to overvalue social gains while undervaluing potential risks, which ultimately leads to unsafe forms of self-presentation.

### Research purpose and significance

Although research on adolescents’ risky online self-disclosure has grown steadily in recent years, significant theoretical divergences and empirical gaps remain in the existing literature.

First, current findings are fragmented and lack theoretical integration. Scholars have examined the antecedents and consequences of adolescents’ risky self-disclosure from diverse perspectives, attempting to uncover its underlying mechanisms and psychological impacts through quantitative approaches (Koutamanis et al., 2015; Sherman et al., 2016; Choi et al., 2019; Yang et al., 2023). However, these studies remain scattered and insufficiently synthesized, leaving no clear consensus on the key predictors and outcomes of adolescents’ risky self-disclosure on social media. Moreover, many studies are limited to specific platforms, cultures, or samples, which restricts the generalizability and consistency of their findings. Therefore, systematically integrating and quantifying previous empirical results is crucial for clarifying the internal logic and mechanisms underlying adolescents’ risky self-disclosure.

Second, the inconsistencies among empirical findings call for further explanation. For instance, Koutamanis et al. (2015) and Sherman et al. (2016) reported contradictory conclusions regarding peer influence: while the former found it to be a facilitating factor, the latter suggested it may play an inhibitory role. Similarly, studies on the effects of age and gender have produced conflicting results (Koutamanis et al., 2015; Paluckaitė and Žardeckaitė-Matulaitienė, 2019; Yang et al., 2023). These discrepancies complicate theoretical interpretations and hinder the practical application of existing findings in risk intervention and media education. One possible explanation lies in contextual moderators such as platform type, cultural background, and social norms, which shape the relationships between antecedents and consequences (Hawk et al., 2015). Hence, a systematic exploration of these moderating variables, including platform characteristics, age groups, and cultural contexts, holds both theoretical and practical value for reconciling inconsistent findings and uncovering underlying mechanisms.

Building upon these gaps, the present study employs a meta-analytic approach to systematically integrate and quantitatively examine the antecedents and outcomes of adolescents’ risky online self-disclosure. Drawing upon the framework of Privacy Calculus Theory (Dinev and Hart, 2006), this study seeks to explain adolescents’ risky self-disclosure through the balance between perceived benefits and perceived costs. Perceived benefits, including peer influence, parental support, and sensation seeking, are compared with perceived costs, such as anxiety, depression, and negative emotions, to explore their interactive effects on adolescents’ risky online behavior. Furthermore, this study investigates the psychological and social consequences of risky self-disclosure, including negative peer feedback, cyberbullying exposure, and psychological stress (Peluchette et al., 2015; Paluckaitė and Žardeckaitė-Matulaitienė, 2019; Pazdur et al., 2025). Moderation analyses are also conducted to examine how platform type (e.g., Instagram, TikTok, WeChat Moments), developmental stage, and cultural background influence these relationships, aiming to explain inconsistencies in prior studies and delineate the contextual boundaries of risky online behavior.

From a theoretical perspective, this study advances the understanding of adolescents’ risky self-disclosure by moving beyond outcome-oriented frameworks that focus solely on harm and instead emphasizing the cognitive and motivational imbalances within the privacy calculus process. By revealing adolescents’ systematic biases in weighing costs and benefits, the study offers a more explanatory model for understanding self-disclosure behavior in digital environments. From a practical perspective, identifying high-risk patterns of self-disclosure and their underlying cognitive distortions can help adolescents enhance their awareness of digital risks and improve self-protection skills. The findings also provide actionable insights for parents, educators, and social media platforms. Specifically, schools and families may design targeted media literacy and psychological counseling programs, while platforms can optimize risk detection algorithms and privacy reminder systems to achieve a dual defense mechanism integrating technological and psychological dimensions.

In sum, by combining meta-analytic synthesis with the integrative lens of Privacy Calculus Theory, this study addresses theoretical and empirical gaps in research on adolescents’ risky online self-disclosure. It deepens the understanding of its formation mechanisms and consequences and offers theoretical and practical implications for promoting adolescents’ online psychological well-being and enhancing risk governance on social media platforms.

## Method

### Protocol and registration

This systematic review followed the Preferred Reporting Items for Systematic Reviews and Meta-Analyses (PRISMA) guidelines. The review protocol was prospectively registered on the PROSPERO International Prospective Register of Systematic Reviews on August 1, 2025 (registration ID: CRD420251117327).

### Literature screening process

Following a comprehensive systematic search, a set of inclusion and exclusion criteria was established to ensure that all selected studies met the research requirements. The review covered studies published between 2015 and 2025 (a ten-year period).

Inclusion criteria were as follows. (1) Participants were adolescents aged between 10 and 21 years who actively engaged in social media platforms and demonstrated self-disclosure behaviors, including sharing personal information, photos, or other online content; (2) Studies were peer-reviewed empirical investigations employing quantitative research designs, including but not limited to cross-sectional surveys, experimental studies, and longitudinal designs; (3) Studies examined risky online self-disclosure as either an independent or dependent variable and reported sample size and correlation coefficients, allowing for the computation of effect sizes; (4) Studies were written in English and available in full text.

Exclusion criteria included. (1) Studies focusing on participants outside the adolescent age range (≥22 years or ≤9 years); (2) Theoretical papers, qualitative studies, review articles, or studies that did not employ quantitative methods; (3) Studies that did not treat risky online self-disclosure as an independent or dependent variable, or that focused exclusively on offline self-disclosure or general internet use without a social media component; (4) Studies that did not report sample size, correlation coefficients, or other convertible statistical data necessary for effect size estimation; (5) Studies not written in English or not available in full text.

### Literature search

The initial literature search for this study was completed in September 2025 to systematically integrate empirical research on adolescents’ risky online self-disclosure. Four major academic databases were searched: PsycINFO, PubMed, Scopus, and Web of Science. To enhance the breadth and rigor of the search strategy and to capture additional relevant studies, the search terms were updated in November 2025 to include “self-harm” and “self-esteem.” The final search strategy combined controlled vocabulary and free-text terms with Boolean operators: (“online self-disclosure” OR “self-disclosure” OR “risky self-disclosure” OR “risky self-presentation” OR “online self-presentation” OR “digital self-disclosure” OR “social media disclosure” OR “personal information sharing” OR “photo disclosure” OR “information disclosure”) AND (adolescent* OR youth OR teenager* OR “young people” OR “high school students”) AND (“cyberbullying” OR “peer feedback” OR “negative feedback” OR “online victimization” OR “online sexual solicitation” OR “self-harm” OR “self-esteem”).

**Figure 1:**
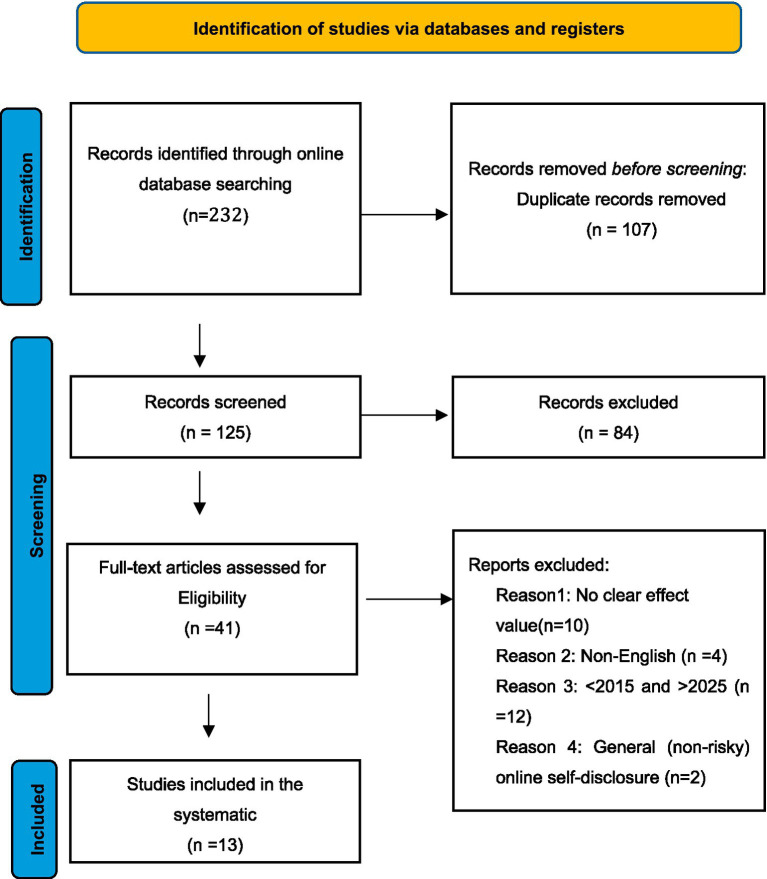
Screening process of research literature.

The screening process followed a systematic procedure. Duplicate records were removed using built-in database tools. Titles and abstracts were then screened to determine relevance. Full texts of potentially eligible studies were subsequently reviewed to evaluate whether they met the inclusion criteria. The search and screening process is illustrated in Figure 1.

The initial search identified 232 records. After removing 107 duplicates, 125 records remained for title and abstract screening. Eighty-four records were excluded due to lack of relevance. The remaining 41 studies underwent full-text review. Twenty-eight studies were excluded at this stage: 10 did not report effect sizes, 4 were not published in English, 12 were outside the 2015–2025 publication period, and 2 addressed general online self-disclosure rather than risky online self-disclosure. Thirteen studies met all inclusion criteria and were retained for the meta-analysis.

### Data coding

To ensure the reliability and accuracy of data coding, the process was independently conducted and cross-checked by multiple researchers. Any discrepancies identified during the coding process were resolved through discussion and consensus to enhance the credibility of the results. All information presented in the review reflects the final coding outcomes agreed upon by all coders. Table 1 summarizes the key data extracted from the included studies, including author, year of publication, variable category, independent variable, dependent variable, type of effect size, original effect value, sample size, country, mean age, and type of social media platform.

**Table 1:** Characteristics of studies included in the meta-analysis.

No.	Author year	Country	N	Mean age	Platform	Variable type	IV	DV	Effect type
1	Aizenkot (2020)	Israel	5,581	12	GSM	Consequence	Risky self-disclosure	Cyberbullying	0.08(β)
2	Babilonová et al. (2024)	Czech Republic	1,095	16.82	GSM	Antecedent (cost)	Depression	Self-disclosure video to strangers	0.315(β)
						Antecedent (cost)	Anxiety	Self-disclosure video to strangers	0.468(β)
						Antecedent (benefit)	Parental support	Self-disclosure video to strangers	−0.23(β)
3	Baumgartner et al. (2015)	Germany	238	14.75	Facebook	Antecedent (cost)	Peer influence	Risky self-disclosure photo	0.47(β)
4	Chang et al. (2021)	Hong Kong	3,772	14.5	GSM	Consequence	Risky sexual disclosure	Suicide ideation	0.29(β)
5	Choi et al. (2019)	South Korea	7,109	14.51	GSM	Consequence	Risky self-disclosure	Cyberbullying	0.095(r)
6	Festl et al. (2019)	Germany	1,033	17	GSM	Consequence	Risky sexual disclosure	Online Sexual Victimization	0.22(β)
7	Hawk et al. (2015)	Amsterdam	471	14.75	SNS	Antecedent (cost)	High narcissism low entitlement	Risky self-disclosure	−0.55(*β*)
8	Hsieh et al. (2023)	Taiwan	19,556	13.77	GSM	Consequence	Risky self-disclosure	UOSS	0.16(β)
9	Koutamanis et al. (2015)	Netherlands	785	12.56	GSM	Antecedent (benefit)	Sensation seeking	Risky self-disclosure photo	0.14(β)
						Antecedent (cost)	Peer influence	Risky self-disclosure photo	−0.15(β)
						Antecedent (cost)	Age	Risky self-disclosure photo	0.02(β)
						Consequence	Risky self-disclosure photo	Negative peer feedback	0.26(β)
10	Paluckaitė and Žardeckaitė-Matulaitienė (2019)	Lithuania	459	14.55	GSM	Antecedent (benefit)	Positive emotion	Risky self-disclosure photo	0.82(β)
						Antecedent (cost)	Negative emotion	Risky self-disclosure photo	−0.13(β)
						Antecedent (cost)	Age	Risky self-disclosure photo	0.09(β)
						Antecedent (cost)	Narcissism	Risky self-disclosure photo	0.25(β)
						Antecedent (cost)	Gender	Risky self-disclosure photo	−0.12(β)
11	Peluchette et al. (2015)	United States and Australia	572	21.1	Facebook	Consequence	Risky self-disclosure	Cyberbullying mild	0.13(r)
						Consequence	Risky self-disclosure	Cyberbullying severe	0.15(r)
12	Sherman et al. (2016)	USA	32	15.5	Instagram	Antecedent (cost)	Peer influence	Risky self-disclosure	2.34(t)
13	Yang et al. (2023)	China	845	18.7	WeChat and Weibo	Antecedent (cost)	Attachment anxiety	Risky self-disclosure	0.093(β)
						Consequence	Risky Self-disclosure	Cyberbullying	0.14(β)

Each row represents a separate effect size. GSM = General social media; IV = independent variable; DV = dependent variable; β = standardized regression coefficient; r = correlation coefficient; t = t-value; *N* = sample size. The categorization of antecedents into benefit-related and cost-related types follows the operationalization of the privacy calculus framework in adolescents’ online risky self-disclosure.

In terms of variable classification, this study adopts Privacy Calculus Theory (PCT) as its theoretical foundation and categorizes variables based primarily on their theoretical functions rather than the statistical direction of their relationships. When risky self-disclosure is treated as the dependent variable (DV), related variables are conceptualized as antecedents. Consistent with the benefit–cost framework of PCT, antecedents are further divided into two categories.

The first category consists of benefit antecedents. These refer to factors that influence adolescents’ perceptions of the benefits of disclosing personal information, which in turn encourage risky self-disclosure by increasing perceived benefits or reducing psychological costs. Variables in this category include sensation seeking, parental support, and positive emotions (Koutamanis et al., 2015; Babilonová et al., 2024). Prior research indicates that adolescents with high levels of sensation seeking are more likely to engage with stimulating and high-risk online content and to participate in risk-taking behaviors both online and offline (Khurana et al., 2019; Pazdur et al., 2025). Based on these findings, individuals with elevated sensation seeking may be more sensitive to potential psychological stimulation, social approval, and peer interaction. Within the PCT framework, sensation seeking can therefore be conceptualized as a psychological and social benefit-driven antecedent of adolescents’ risky online self-disclosure.

The second category consists of cost antecedents. These variables influence adolescents’ perceptions of the risks or potential losses associated with disclosure. Existing studies suggest that emotional vulnerability factors such as anxiety, depression, and negative affect may heighten sensitivity to potential online harms by reducing adolescents’ capacity to cope with risks (Babilonová et al., 2024; Yang et al., 2023). Social contextual pressures, including peer influence and power hierarchies, can also amplify social evaluation concerns and psychological burden, thereby increasing perceived potential losses in disclosure decisions (Hawk et al., 2015; Baumgartner et al., 2015; Sherman et al., 2016). To further enrich the classification system, age and gender were also included as potential cost antecedents. Theoretical and empirical evidence suggests that younger adolescents, who often exhibit lower levels of risk perception, are more likely to engage in risky online disclosure (Koutamanis et al., 2015). Regarding gender, studies by Paluckaitė and Žardeckaitė-Matulaitienė (2019) and Koutamanis et al. (2015) found that girls are more likely than boys to engage in risky self-disclosure through images or photographs. These categorizations align with the functional definition of cost antecedents in PCT, in which such factors increase perceptions of potential negative consequences associated with disclosure.

Correspondingly, when adventurous self-disclosure is treated as the independent variable, its related variables are classified as outcomes that reveal the potential psychological, social, or behavioral consequences of disclosure.

In addition, to enhance the robustness of the classification, this study supplements the theoretical categorization by consulting existing literature on the predominant empirical tendencies of each variable, such as its documented statistical relationship with adventurous self-disclosure. When notable discrepancies arise between theoretical assumptions and empirical evidence, we revisit theoretical definitions and carefully adjust variable placement, ensuring consistency with theoretical logic and adequate empirical support.

### Data extraction and analysis

In this study, all quantitative data were processed and analyzed using the latest meta-analysis packages in RStudio (Version 2025.1), including *metafor* (Viechtbauer, 2010), *meta* (Schwarzer et al., 2015), and *dmetar* (Harrer et al., 2021). To ensure comparability of effect sizes across studies, all original effect values were converted into Pearson’s correlation coefficients (r) and their Fisher’s z-transformed values (z) prior to analysis (Borenstein et al., 2021).

Because the statistical indicators reported across studies varied (e.g., *r*, *β*, *t*), standardization of effect sizes was necessary before aggregation. Following the recommendations of Borenstein et al. (2021) and Lipsey (2001), all effect sizes were directionally aligned, and variances were weighted by sample size to enhance the precision of the overall estimates.

For effect size synthesis, this study adopted a random-effects model to estimate the pooled effects of the antecedents and outcomes of adolescents’ risky self-disclosure. Unlike the fixed-effects model, the random-effects model assumes that true effects vary across studies, thereby capturing potential heterogeneity arising from differences in samples, platforms, and cultural contexts (Borenstein et al., 2010). Heterogeneity was assessed using the Q statistic and I^2^ index, where a significant Q value (*p* < 0.05) indicates substantial heterogeneity among studies, and I^2^ values ranging from 0 to 100% reflect its magnitude—values exceeding 50% typically suggest moderate to high heterogeneity (Higgins et al., 2003).

To further explore the sources of heterogeneity and examine potential contextual influences, subgroup analyses and meta-regression analyses were conducted. Subgroup analyses compared effect size differences across variables such as platform type, cultural background, and age group, while meta-regression modeled the moderating effects of continuous or categorical variables on the relationships involving adolescents’ risky self-disclosure (Thompson and Higgins, 2002). Through this set of analyses, the study sought to identify systematic variations and underlying mechanisms of adolescents’ risky self-disclosure across different sociocultural and platform contexts.

## Results

### Overall effect sizes and heterogeneity tests

To systematically examine the overall relationships between adolescents’ risky online self-disclosure and its antecedent and outcome variables, three separate random-effects models were constructed based on variable types (antecedent benefit, antecedent cost, and consequence). Effect sizes were integrated using the restricted maximum likelihood (REML) estimation method. All effect values were expressed as Fisher’s z, which were subsequently converted to correlation coefficients (r) for ease of interpretation.

**Figure 2:**
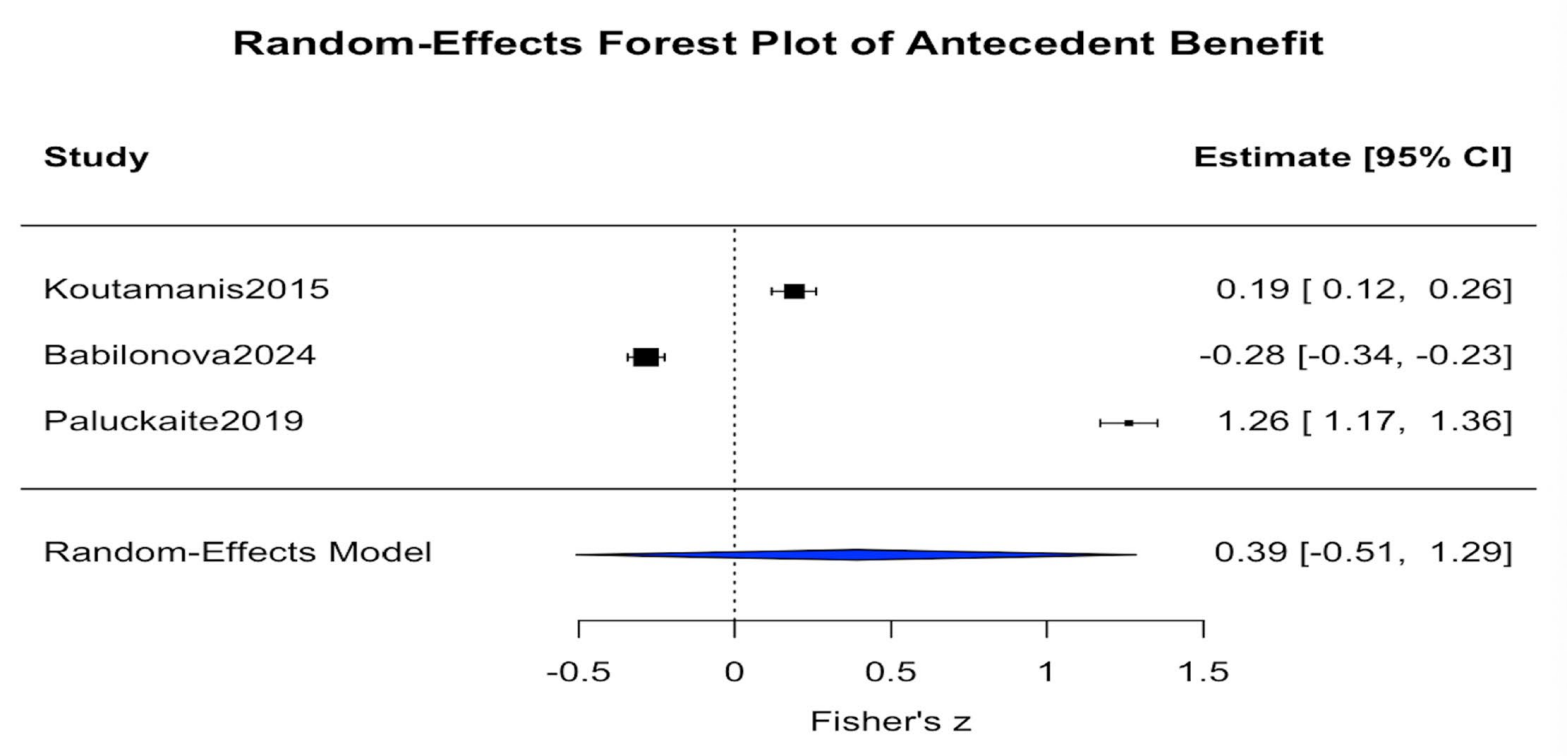
Forest plot of antecedent benefit.

The analysis of benefit-related antecedents, including parental support, positive emotions, and sensation seeking, consisted of k = 3 independent studies. Results from the random-effects model (see Figure 2) showed an average Fisher’s z of 0.389, which corresponds to an average correlation coefficient of approximately r = 0.37. The standard error was SE = 0.458, with a 95 percent confidence interval ranging from −0.51 to 1.29, and the *p* value was 0.396. The heterogeneity statistic indicated an I^2^ value of 99.78 percent, suggesting a very high level of heterogeneity across studies. Overall, due to the limited number of studies and the extremely high heterogeneity, the results did not reach statistical significance and should therefore be interpreted with caution. This finding suggests substantial variation in effect sizes across studies and indicates that the motivational influence of perceived benefits on adventurous self-disclosure may depend on the interactions among cultural context, sample characteristics, and the features of specific social media platforms.

**Figure 3:**
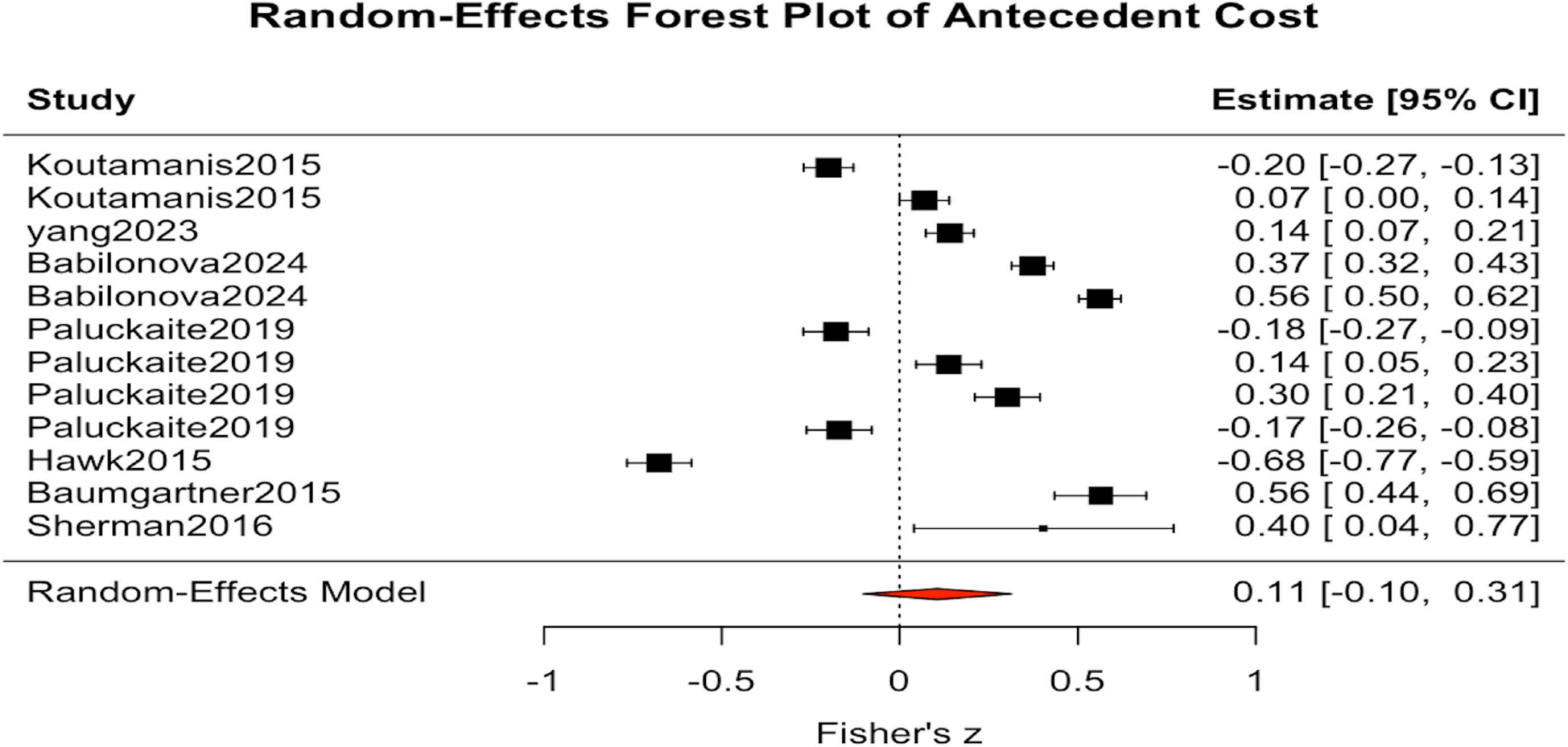
Forest plot of antecedent cost.

Similarly, Figure 3 presents the analysis of cost-related antecedents, such as depression, anxiety, negative emotions, and peer pressure, which included k = 12 studies. The results showed an average Fisher’s z of 0.106, corresponding to an average correlation coefficient of approximately r = 0.11. The standard error was SE = 0.106, with a 95 percent confidence interval ranging from −0.10 to 0.31, and the *p* value was 0.318. The heterogeneity statistic indicated an I^2^ value of 98.71 percent, suggesting substantial variation across studies. The results were not statistically significant; therefore, no firm conclusion can be drawn at this stage. This finding suggests that adolescents’ engagement in online adventurous self-disclosure is not solely driven by risk-related psychological traits. Instead, it is likely shaped by the combined influence of multiple factors, including developmental stage, socialization environment, cultural norms, and emotional needs (Stoilova et al., 2019; Valkenburg and Piotrowski, 2017).

**Figure 4:**
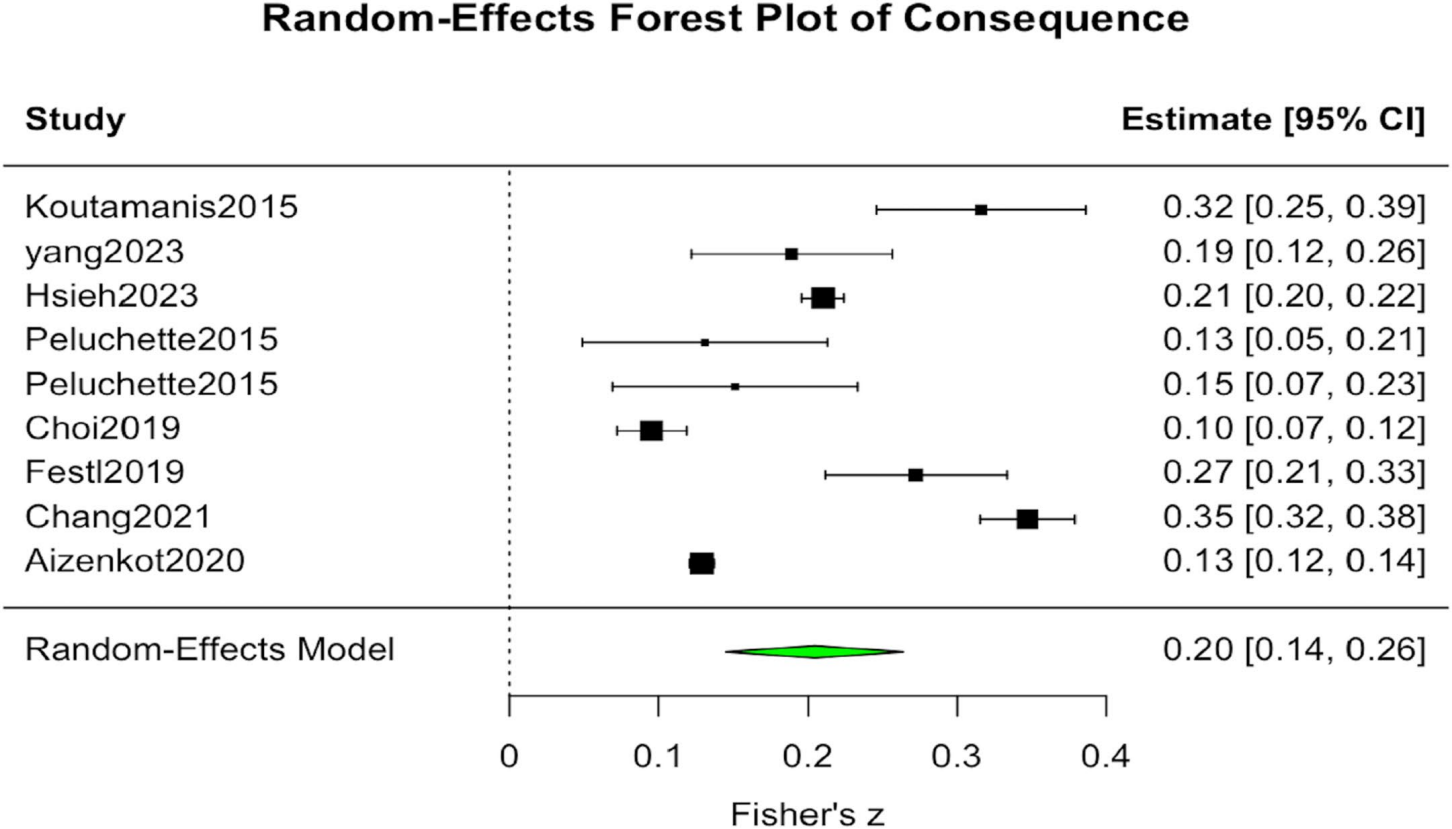
Forest plot of consequence.

In contrast, the analysis of outcome variables included k = 9 studies. Results from the random-effects model showed an average Fisher’s z of 0.205, corresponding to an average correlation coefficient of approximately r = 0.202. The standard error was SE = 0.030, with a 95 percent confidence interval ranging from 0.145 to 0.264, and the *p* value was below 0.001. The heterogeneity statistic indicated an I^2^ value of 97.94 percent, suggesting considerable variation across studies. The meta-analysis results demonstrate a significant positive association between adolescents’ adventurous self-disclosure and psychological and social outcomes such as negative peer feedback, cyberbullying, or UOSS. Although heterogeneity remains high across studies, with I^2^ values exceeding 97 percent, the direction of the effect is consistent. This pattern suggests that the relationship is relatively robust across different research contexts (see Figure 4).

Overall, the random-effects model reveals a dual pattern in adolescents’ online adventurous self-disclosure. The effects of antecedents, whether benefit-related or cost-related, exhibit substantial variability, whereas the effects of outcomes appear consistent across studies. This finding highlights the complexity and context-dependent nature of the behavior. It also suggests that future research should explore how cultural context, platform characteristics, and individual differences jointly moderate this process in order to advance understanding of the mechanisms and psychological consequences underlying adolescents’ online adventurous self-disclosure (Valkenburg and Piotrowski, 2017).

### Publication bias and robustness analysis

**Figure 5:**
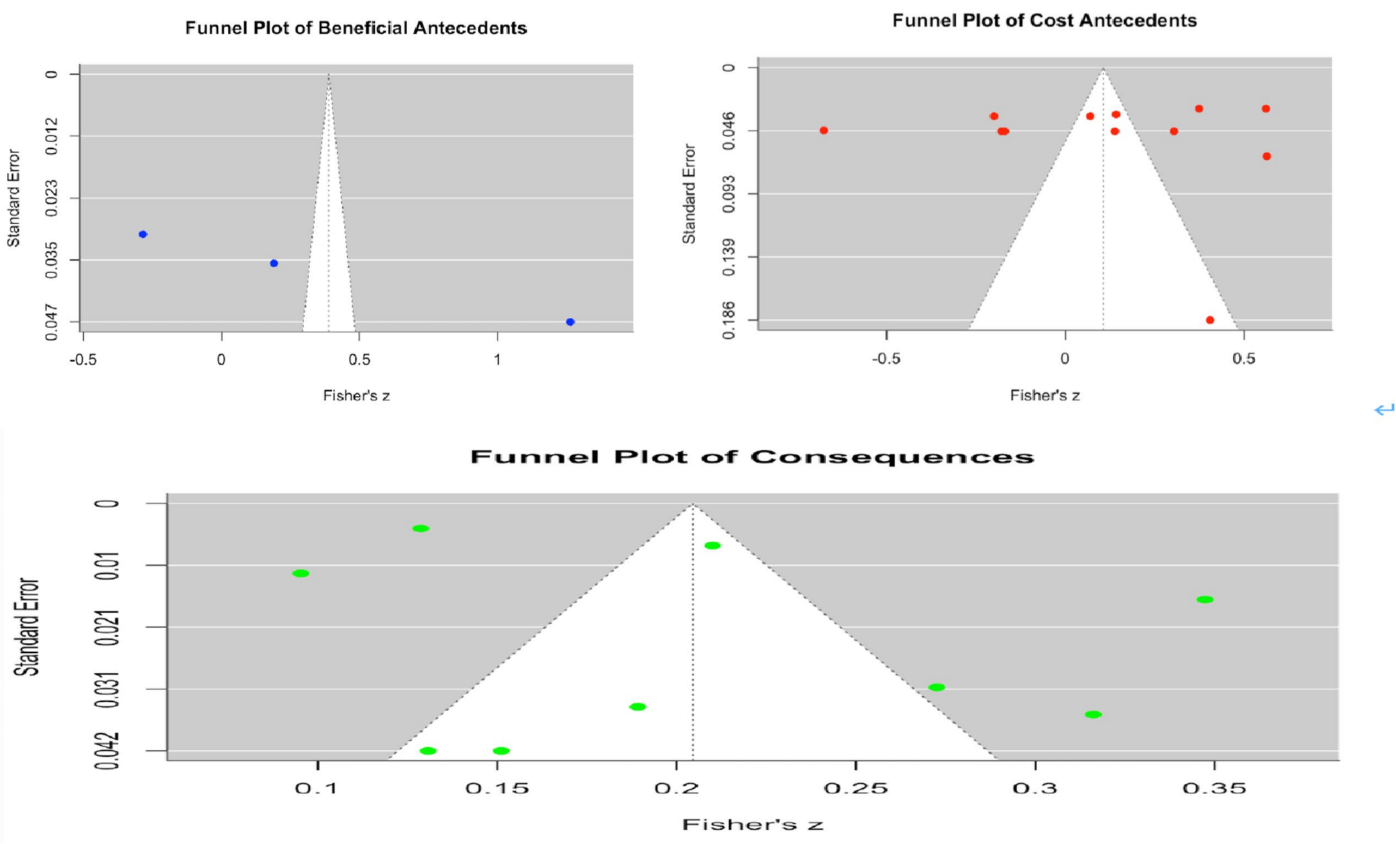
Funnel plots of three meta-analytic models: beneficial antecedents, cost antecedents, and consequence.

To examine the possibility of publication bias, this study conducted funnel plot analyses and Egger’s regression tests for the three meta-analytic models: benefit-related antecedents, cost-related antecedents, and consequences. As shown in Figure 5, the funnel plot for the benefit-related antecedent model displays a slight degree of asymmetry. The Egger’s test indicated significant bias, with z = 27.75 and *p* < 0.001, suggesting that the overall effect of benefit-related antecedents on adventurous self-disclosure may have been inflated by the influence of certain studies. In contrast, the funnel plot for the cost-related antecedent model appears largely symmetrical (Figure 5). The Egger’s test was not significant, with z = 0.70 and *p* = 0.4846, indicating a relatively low risk of publication bias. Similarly, the funnel plot for the consequence model (Figure 5) displays a symmetrical distribution. The Egger’s test was not significant, with z = 0.32 and *p* = 0.7522, suggesting that the relationship between adventurous self-disclosure and its outcomes is relatively robust and does not appear to be affected by substantial publication selection bias.

**Figure 6:**
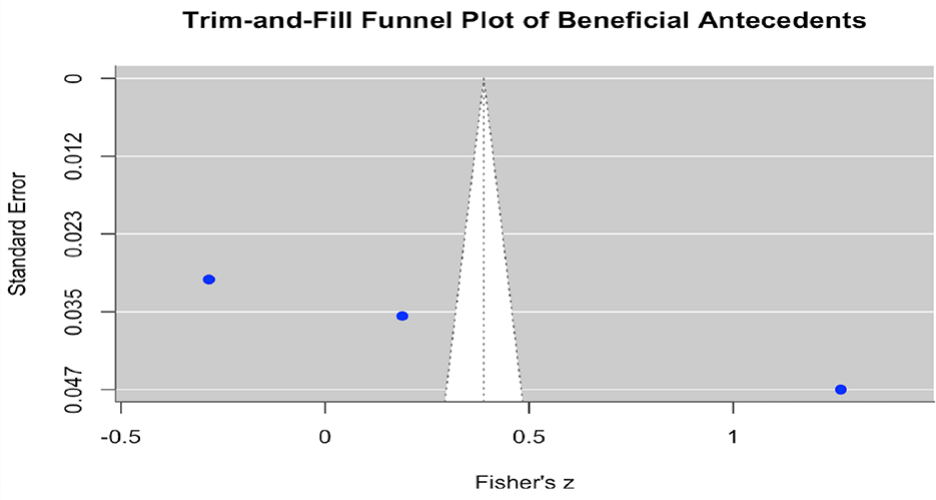
Trim-and-fill analysis results for the three meta-analytic models: Beneficial antecedents.

In addition, the Trim-and-Fill analysis further supported these findings (Figure 6). Only the benefit-related antecedent model yielded an estimate of two potentially missing studies, yet the adjusted overall effect size remained consistent with the original result. Neither the cost-related antecedent model nor the outcome model required the addition of missing studies, indicating a high level of robustness in these results.

Beyond publication bias assessment, we also conducted a basic evaluation of the characteristics of the included studies to provide an initial indication of research quality. This assessment considered sample size, study design, and measurement reliability for each study. Because the number of included studies was limited (*n* = 13), the evaluation was relatively brief and does not constitute a comprehensive systematic risk-of-bias assessment. However, it offers useful contextual information for interpreting the meta-analytic findings and highlights potential limitations in the included studies. Overall, publication bias was primarily observed in the benefit-related antecedent model, whereas the effect sizes for the cost-related antecedent model and the outcome model were statistically stable. These results suggest that the conclusions of this meta-analysis demonstrate a reasonable degree of robustness.

To provide preliminary information regarding the risk of bias at the study level, this research conducted a basic quality assessment of the 13 studies included in the final analysis. The assessment dimensions consisted of sample size, research design, reliability of measurement instruments, and the clarity of the operationalization of risky online self-disclosure. Each dimension was rated as Low, Medium, or High. In consideration of the reasonable variation in sample size requirements across different research designs, experimental studies could still be rated as medium or high quality even when their sample sizes were relatively small, whereas cross-sectional or survey-based studies were evaluated according to conventional sample size standards. The overall quality rating was determined by synthesizing all four dimensions. This quality assessment is intentionally concise and is designed to assist in interpreting the meta-analytic findings and to highlight potential methodological limitations within the included studies (see Appendix A).

### Moderation analysis: mean age and sample size

To examine whether participant characteristics influenced the associations between adolescents’ risky online self-disclosure and its antecedents or consequences, meta-regression analyses were conducted using mean age and sample size (log N) as moderator variables.

To further explore potential sources of between-study heterogeneity, mean age (Mean Age) and sample size (log N) were entered as continuous moderators in three separate meta-regression models corresponding to antecedent benefit, antecedent cost, and consequences (see Appendix B).

The results indicated that mean age did not exert a significant moderating effect in any of the three models. For the benefit-related antecedents, the regression coefficient was *β* = −0.12 with *p* = 0.72. For the cost-related antecedents, the coefficient was β = 0.08 with *p* = 0.20. For the outcomes, the coefficient was β = −0.01 with *p* = 0.47. Although the cost-related antecedent model accounted for approximately 5.7 percent of the between-study heterogeneity (R^2^ = 5.70 percent), the effect did not reach statistical significance. This suggests that adolescents’ mean age has a limited influence on the relationships involving the antecedents and outcomes of adventurous self-disclosure. One likely explanation is that existing studies tend to focus on a relatively narrow adolescent age range, primarily mid- to late adolescence, which may not provide sufficient variability to produce meaningful cross-study differences.

In contrast, sample size exhibited a significant moderating effect in the model of benefit-related antecedents. The results showed that studies with larger samples reported weaker associations between benefit-related antecedents such as parental support and sensation seeking and adventurous self-disclosure, with β = −1.80 and *p* < 0.001. This moderator accounted for approximately 98.7 percent of the residual heterogeneity (R^2^ = 98.73 percent). However, this effect was not significant in either the cost-related antecedent model (β = −0.06, *p* = 0.66) or the outcome model (β = −0.01, *p* = 0.51). These findings suggest that studies with large samples may estimate the association between positive antecedents and adventurous self-disclosure more accurately and consistently, whereas smaller studies may underestimate the effect due to limited statistical power or inconsistencies in measurement.

Taken together, the moderation analysis indicates that study-level characteristics such as sample size provide greater explanatory power for effect size variability than individual-level characteristics such as age. The significant influence of sample size underscores the importance of research design and methodological rigor in accurately identifying the true relationships underlying adolescents’ online adventurous self-disclosure.

## Subgroup analysis: country and platform type

To further examine potential differences in adolescents’ online adventurous self-disclosure across cultural contexts and social media environments, this study conducted exploratory subgroup analyses based on country and platform type. It should be noted that the number of studies in some subgroups was limited, especially cases in which k = 1. Therefore, the findings presented in this section should be understood as indicative trends that may guide future research rather than as definitive causal or cross-cultural conclusions (see Appendix C).

### Country differences

The country-level subgroup analyses covered benefit-related antecedents, cost-related antecedents, and outcome variables. Table 2 summarizes the number of studies representing each country or region to clarify the evidentiary foundation of each subgroup.

**Table 2:** Subgroup distribution of included studies by country/region.

Country/region	k	s	Study sources
Netherlands	5	2	Koutamanis et al. (2015) and Hawk et al. (2015)
China	2	1	Yang et al. (2023)
Czech Republic	3	1	Babilonová et al. (2024)
Lithuania	5	1	Paluckaitė and Žardeckaitė-Matulaitienė (2019)
Germany	3	2	Baumgartner et al. (2015) and Festl et al. (2019)
USA	1	1	Sherman et al. (2016)
USA & Australia (Mixed)	2	1	Peluchette et al. (2015) *
Taiwan	1	1	Hsieh et al. (2023)
South Korea	1	1	Choi et al. (2019)
Hong Kong	1	1	Chang et al. (2021)
Israel	1	1	Aizenkot (2020)

*k* = number of effect sizes; *s* = number of studies. * Refers to the study including a mixed sample (United States and Australia).

For benefit-related antecedents, the Netherlands, the Czech Republic, and Lithuania each contained only one study (k = 1). Because of the limited sample size, heterogeneity and significance tests were not conducted. Although the evidence base is small, these findings underscore the scarcity of cross-cultural research in this area and the uneven geographical distribution of existing studies. This pattern points to a substantial cross-cultural research gap in the field.

For cost-related antecedents, effect sizes varied across countries, and the level of heterogeneity was relatively high, with I^2^ values ranging from 94 percent to 96 percent. Specifically, studies from the Czech Republic (k = 2) demonstrated a significant positive association between cost-related antecedents and adventurous self-disclosure (r = 0.47, *p* < 0.001). In contrast, studies from the Netherlands (k = 3, r = −0.06, *p* = 0.63) and Lithuania (k = 4, r = 0.02, *p* = 0.84) did not show significant associations. Other countries, including China, Germany, and the United States, were not included in the statistical analysis because each contributed fewer than two studies. Overall, the cross-cultural variation in cost-related antecedents appears relatively pronounced, suggesting that these effects may be shaped by cultural and contextual conditions. However, due to the limited number of available studies, these findings should be interpreted as preliminary trends.

For outcome variables, only the combined subgroup representing the United States and Australia (k = 2) met the criteria for subgroup analysis. The results showed a significant positive relationship between adventurous self-disclosure and its consequences (r = 0.14, *p* < 0.001) with low heterogeneity (I^2^ = 0 percent). This indicates that, within the available data, the consequences of adventurous self-disclosure display a certain degree of consistency across cultural contexts. Other countries, including the Netherlands, China, Taiwan, South Korea, Germany, Hong Kong, and Israel, were not included in the subgroup analysis because each contributed only a single study (k < 2).

Overall, the country-level subgroup analysis reveals structural characteristics of the existing literature. Cost-related antecedents exhibit notable cultural sensitivity, whereas benefit-related antecedents and outcome variables cannot be interpreted conclusively due to limited sample size. The evident shortage of cross-cultural research highlights an important gap in the field and suggests that future studies should prioritize multi-country and cross-cultural comparative designs.

### Platform differences

The platform subgroup analysis covered antecedent benefit, antecedent cost, and consequence variables to examine differences in risky self-disclosure across various social media platforms. Table 3 presents the distribution of studies by platform.

**Table 3:** Subgroup distribution of included studies by platform.

Platform	k	s	Study sources
General social media	17	8	Koutamanis et al. (2015), Babilonová et al. (2024), Paluckaitė and Žardeckaitė-Matulaitienė (2019), Hsieh et al. (2023), Choi et al. (2019), Festl et al. (2019), Chang et al. (2021), and Aizenkot (2020)
Instagram	1	1	Sherman et al. (2016)
Facebook	3	2	Peluchette et al. (2015)* and Baumgartner et al. (2015)
WeChat / Weibo	2	1	Yang et al. (2023)
SNS	1	1	Hawk et al. (2015)

*k* = number of effect sizes; *s* = number of studies. * Refers to the study including a mixed sample (United States and Australia).

The results show that antecedent benefit included studies with the “General social media” category (k = 3). The effect size was r = 0.39, although it was not statistically significant (*p* = 0.40), and heterogeneity was extremely high (I^2^ = 99.78 percent). This indicates substantial methodological and measurement variability across existing studies. Other platforms, including WeChat, Weibo, SNS, Facebook, and Instagram, had fewer than two studies and were therefore excluded from the analysis. For antecedent cost, eight studies were available under “General social media,” with an effect size of r = 0.11 and a non-significant *p* value of 0.26. Heterogeneity again remained high (I^2^ = 98.21 percent). Other platforms contained only one study and were not subjected to statistical analysis. These results suggest that the associations between cost-related antecedents and risky self-disclosure vary across platforms and that the existing evidence base remains limited.

In contrast, the consequence variable showed a significant positive association on the platforms that could be analyzed. The “General social media” platform produced an effect size of r = 0.21 (*p* < 0.001, I^2^ = 99.18 percent). The Facebook platform produced an effect size of r = 0.14 (*p* < 0.001, I^2^ = 0 percent). These findings indicate a degree of consistency in the consequences of risky self-disclosure on these platforms. Other platforms, such as Instagram, Snapchat, WeChat, and Weibo, were excluded from analysis due to insufficient study numbers (k < 2). Overall, the platform subgroup analysis highlights structural characteristics of the current literature. Most studies are concentrated in the category of general social media rather than platform-specific contexts. Both antecedent benefit and antecedent cost remain unstable across platforms, whereas the consequence variable exhibits a consistent positive pattern on platforms with sufficient data. These observations suggest that future research should expand to a wider range of platforms to more fully understand the cross-platform characteristics of adolescents’ online risky self-disclosure.

The platform subgroup analysis indicates that adolescents’ risky self-disclosure shows some degree of variation across platforms. Among the platforms that could be statistically analyzed, such as General social media and Facebook, the consequences of disclosure demonstrated significant positive associations that ranged from r = 0.14 to r = 0.21 with *p* values below 0.001. This suggests that disclosure outcomes exhibit relative stability on these platforms. Other platforms, including Instagram, Snapchat, WeChat, and Weibo, could not be analyzed due to the limited number of studies, which makes it impossible to draw firm conclusions. Overall, platform structures and social norms may shape adolescents’ motivations and behaviors related to risky self-disclosure. This highlights the need for future research to expand cross-cultural and cross-platform examinations of these behaviors (Towner et al., 2022; Marciano and Viswanath, 2023; Balamurugan and Vijayarani, 2025).

## Discussion

### Major findings and research implications

It should be noted at the outset that the number of primary studies included in this research is relatively limited, and for some antecedent variables, effect sizes are based on very few studies. Therefore, the theoretical interpretations and practical implications of the main findings should be understood within a cautious and exploratory framework. On this basis, although the overall effects were not statistically significant, the results of this study provide valuable insights for understanding the complex mechanisms underlying adolescents’ risky online self-disclosure and potential directions for intervention.

This study systematically integrated empirical evidence on the antecedents and outcomes of adolescents’ risky online self-disclosure through meta-analysis, identifying several theoretically informative patterns. Overall, the results indicate that neither variables typically classified as benefit antecedents, such as sensation seeking, positive affect, and parental support, nor those classified as cost antecedents, including negative affect, peer influence, anxiety, and depression, showed statistically significant average effects on adolescents’ risky online self-disclosure. Moreover, the results across studies exhibited a high degree of heterogeneity. This finding suggests that attempting to explain adolescents’ risky self-disclosure behavior solely through a single psychological factor or simple linear causal pathways may risk oversimplification. Differences in effect sizes across studies are likely influenced by a combination of factors, including measurement methods, sample characteristics, and research contexts.

To better understand the non-significant overall effects, a more cautious interpretive perspective is necessary. It is important to note that the antecedent constructs included in the current meta-analysis mainly focus on a limited set of variables, such as sensation seeking, emotional states, and parental support, which may not fully capture the motivational structure of adolescents’ online self-disclosure. Therefore, the absence of significant average effects should not be interpreted as evidence that these factors have no influence on risky self-disclosure. Rather, it likely reflects the highly contextualized and multi-determined nature of such behaviors. For example, factors such as peer interaction patterns, platform-specific technical features, immediate social feedback mechanisms, and short-term emotional fluctuations, as reported in previous studies (Baumgartner et al., 2015; Hawk et al., 2015; Yang et al., 2023), were not consistently or systematically measured across studies, which may weaken the stability of linear predictions based on the traditional benefit–cost framework in cross-study integration. In other words, existing evidence suggests that adolescents’ risky self-disclosure may be embedded within more complex mechanisms that have not yet been fully captured, rather than simply resulting from a lack of risk or benefit motivation.

At the same time, the high heterogeneity observed in this study further indicates that the relationship between antecedent variables and risky self-disclosure may be moderated by contextual and methodological factors. To explore this possibility, this study conducted preliminary analyses of potential moderators using information available from the original studies, including mean age, platform type, country or region, and sample size. Although overall moderation effects were limited, some results suggested that study context could influence effect direction or magnitude to some extent. For instance, antecedent effects varied across different platform types, such as image-dominated versus text-dominated platforms, and across samples from different cultural backgrounds, which is consistent with the contextual sensitivity implied by high heterogeneity. However, due to substantial inconsistencies in variable operationalization and reporting across the original studies, the available information for moderation analyses was limited. Therefore, these findings should be considered preliminary and require further validation in future research through standardized measurement frameworks, expanded antecedent constructs, and improved data transparency.

Beyond theoretical interpretation, the results of this study also offer limited but insightful implications for educational practice, family digital parenting, and platform governance. First, the absence of stable predictive effects of individual psychological or family factors, such as sensation seeking, positive affect, or parental support, suggests that current school-based online safety education programs that primarily focus on general risk awareness or emotion regulation training (Thorn, 2024) may have context-dependent effectiveness in reducing risky self-disclosure. Future educational interventions may benefit from incorporating context-sensitive and simulation-based teaching strategies that closely reflect real online social interactions, helping adolescents develop the ability to identify and judge privacy boundaries, social feedback, and potential risks in specific social scenarios (Thorn, 2024; Kilmer et al., 2024; National Telecommunications and Information Administration, 2024).

Second, at the family level, the lack of a stable significant relationship between parental support and risky self-disclosure indicates that emotional support alone may be insufficient as a protective factor against adolescents’ online risk behaviors. Instead, parental practices in digital contexts, such as guidance, rule setting, and co-use strategies (Livingstone and Helsper, 2008; Nikken and Jansz, 2014), may warrant greater attention in future research and interventions. Family-level interventions could therefore explore a comprehensive digital parenting model that balances support, supervision, and communication, while continuing to emphasize the importance of emotional support. The effectiveness of such interventions still requires further high-quality empirical validation (Lou et al., 2024; Geurts et al., 2024).

Finally, from the perspective of platform governance and public policy, the high heterogeneity and context-dependent effects observed in this study suggest that adolescents’ risky self-disclosure is not solely determined by individual characteristics but is also deeply embedded in platform structures and social feedback mechanisms. This implies that interventions relying solely on enhancing user risk awareness or providing general risk warnings may have limited effectiveness. Where feasible, social media platforms could explore structural measures to protect minors, including default privacy settings, content visibility controls, restrictions on interactions with strangers, and transparency in recommendation algorithms (Eltaher et al., 2025; Chawki, 2025). The effectiveness of tiered protection systems and platform compliance mechanisms should be systematically evaluated in future empirical research.

### Interpretation of moderation effects

From a developmental perspective, this study did not find that age consistently moderates the antecedent–outcome associations of adolescents’ online risky self-disclosure. This pattern may suggest that, although adolescents differ in cognitive maturity and self-regulatory capacities as they grow older (Valkenburg and Piotrowski, 2017), they are exposed to highly homogeneous social media environments in which social norms, peer pressure, and expectations for self-presentation tend to be similar across age groups (Livingstone and Blum-Ross, 2020). As a result, risky self-disclosure during adolescence may be shaped more strongly by the combined influence of social contexts and psychological motivations than by age differences alone. This interpretation also indirectly supports the view that adolescents’ online self-disclosure demonstrates a certain degree of “developmental-stage stability.”

In contrast, methodological characteristics at the study level, particularly sample size, offer a more meaningful explanation for variations in effect sizes. Differences in sample size influence not only statistical power but also the stability and reproducibility of effect estimates. Prior methodological research has shown that small-sample studies are more susceptible to measurement error, sampling bias, and contextual noise, which can result in fluctuating or even inconsistent effect directions. Larger samples, however, are more likely to yield robust and generalizable estimates (Cohen, 1992; Schmidt and Hunter, 2015). Therefore, variations in sample size across studies are likely to be an important source of divergence in existing findings.

This result further highlights the critical role of methodological factors in research on adolescents’ social media behaviors. Beyond sample size, platform type, measurement instruments, and research design may also jointly influence the stability and comparability of effect estimates. Future research should ensure adequate sample sizes while also advancing cross-platform and cross-cultural comparative studies and promoting greater standardization of measurement tools in order to reduce study-level heterogeneity and enhance the external validity of conclusions.

Overall, the moderation analysis indicates that study-level design characteristics may play a more pivotal role than individual-level developmental variables, such as mean age, in accounting for differences in observed effects. This conclusion aligns with Valkenburg and Piotrowski’s (2017) Social Media Developmental Model, which proposes that adolescents’ online behavior patterns remain relatively stable during this developmental period, whereas research contexts and methodological conditions are major sources of variability in empirical results.

### Country and platform differences

This study examined differences in adolescents’ risky online self-disclosure at the country and platform levels, focusing on variations in internet usage contexts, digital norms, and platform functionalities. Given the highly uneven distribution of data across countries and platforms, some subgroups included only a single study, and the findings should therefore be interpreted as indicative trends. These results suggest that national and platform-level factors may play a role in shaping adolescents’ risky online self-disclosure, with the underlying mechanisms explored in the following sections.

#### Country differences

Differences at the national level suggest that adolescents’ risky online self-disclosure is not solely an individual psychological behavior but is jointly shaped by country-level internet usage contexts, digital norms, and social support structures. Although empirical evidence for cross-national comparisons remains limited, variations in effect directions across different national samples can be partially interpreted within the framework of national internet environments and adolescents’ online usage contexts.

First, cross-national evidence regarding benefit antecedents remains extremely limited. Nevertheless, these results highlight a key issue: comparative research examining how anticipated benefits drive adolescents’ risky self-disclosure across different national contexts is clearly lacking. This gap makes it difficult to answer why adolescents perceive risky online self-disclosure as a potentially rewarding behavior in varying internet environments and platform usage contexts.

Second, cost antecedents show some interpretable differences across national samples. Findings indicate that the effects of cost antecedents vary significantly between countries. In the Czech Republic, for example, cost antecedents are positively associated with risky self-disclosure, suggesting that negative emotions, such as loneliness and stress, may encourage adolescents to engage in potentially risky online disclosure. This phenomenon may relate to the frequent use of the internet by Czech adolescents as a space for emotional expression and peer interaction (Bedrošová et al., 2018; Šablatúrová et al., 2022; Babilonová et al., 2024). Previous studies have also found that Czech adolescents exhibit a strong tendency toward exploratory internet use (Mýlek et al., 2023), which may strengthen the link between psychological stress and online disclosure behaviors.

In contrast, results from the Netherlands and Lithuania were not statistically significant. In these countries, internet use is often accompanied by clearer privacy norms and risk education, and adolescents may have a more accurate understanding of the potential consequences of online disclosure. Under these conditions, negative emotions do not necessarily translate into risky online self-disclosure. This difference may partly reflect the availability of offline support through family, peers, schools, or other social channels, which reduces reliance on the internet for emotional expression (Valkenburg and Peter, 2013; Gao et al., 2023; CH-Wang et al., 2023). Therefore, the mechanism in which cost factors drive disclosure is not clearly observed in these countries. It is important to note that the Czech Republic is not inherently disadvantaged in terms of macro-level internet infrastructure, but adolescents there rely more heavily on the internet for emotional communication and social exploration (Smahel et al., 2020). This contextual difference in usage patterns may partially explain the different directions of cost antecedent effects.

Thus, national differences in cost antecedents likely reflect not only variations in cultural values but also structural differences in country-level internet ecosystems, adolescents’ daily usage habits, and alternative support channels.

Finally, limited evidence regarding outcome variables shows a relatively consistent pattern. Only the combined sample from the United States and Australia met statistical testing criteria, revealing a significant positive association between risky self-disclosure and negative outcomes. This finding suggests that in countries with widespread internet access and highly routine platform use, the consequences of risky disclosure, such as cyberbullying, may follow a relatively consistent pattern (Twenge et al., 2019).

#### Platform differences

Platform subgroup analyses further indicated that adolescents’ risky self-disclosure varies across social media platforms. Platforms suitable for statistical analysis, such as general social media (GSM) and Facebook, showed significant positive associations with disclosure outcomes. This suggests that the interaction structures, feedback mechanisms, and content presentation formats of these platforms may enhance the positive feedback adolescents receive from risky disclosure.

Although GSM refers to unspecified platforms, risky disclosure within this category was also positively associated with outcomes, indicating that adolescents may achieve social rewards or psychological satisfaction even on non-specific platforms. This may reflect common social motivations across platforms, such as sensation seeking, self-enhancement, and peer influence. Facebook’s real-name structure, strong interaction chains, and explicit feedback systems, including comments and likes, may amplify the social rewards of risky disclosure, making it more predictable and consistent in users’ experiences. These findings are consistent with the “relational disclosure pattern” proposed by Marciano and Viswanath (2023), which suggests that social monitoring and identity constraints in familiar networks reduce the likelihood of high-risk disclosure.

In contrast, platforms such as Instagram, WeChat, Weibo, and Snapchat could not be compared statistically due to limited sample sizes. Nevertheless, the distinct design logics, interaction cultures, and visibility structures of these platforms suggest they may influence risky disclosure in different directions. For instance, visual-oriented interactions, ephemeral content, close-knit peer networks, and platform regulatory mechanisms may all play a role in adolescents’ risk assessment.

An integrated analysis across countries and platforms indicates that online environments, platform structures, and social interaction need jointly shape the differentiated patterns of adolescents’ risky online self-disclosure. Although some subgroups were constrained by limited sample sizes, the study reveals potential contextual and structural mechanisms underlying risky self-disclosure by drawing on perspectives from media ecology and platform functionality.

### Theoretical integration and implications

This meta-analysis systematically synthesized empirical studies on adolescents’ online risky self-disclosure published between 2015 and 2025, developing an integrative framework encompassing antecedents, outcomes, and moderating factors. The findings revealed that both benefit and cost antecedents showed inconsistent predictive effects, whereas the overall effects of consequences were relatively robust. Sample size emerged as a significant moderator in the antecedent benefit model, and substantial national context and platform-based variations were observed. Based on these results, this study extends existing theoretical perspectives on adolescent media use and self-disclosure in three major ways.

First, the study broadens the theoretical boundaries of research on adolescents’ media use and its effects. Previous frameworks, such as the Differential Susceptibility to Media Effects Model (DSMM; Valkenburg and Peter, 2013) and the Social Media Use and Well-Being Framework (Valkenburg, 2022), primarily focused on how the duration and intensity of media use influence psychological outcomes. By contrast, this meta-analysis emphasizes the *risky nature* of self-disclosure and its socio-psychological consequences, highlighting adolescents’ self-presentation and exploratory behaviors in social media contexts. The results support the concept of situated media effects (Slater, 2007), suggesting that risky self-disclosure is not solely the product of individual traits but also shaped by media environments and cultural contexts.

Second, this study used country and platform level subgroup analyses to reveal multidimensional differences in adolescents’ risky online self-disclosure, suggesting that country-level internet usage contexts, digital norms, and social support structures may interactively shape disclosure behaviors. At the national level, variations in adolescents’ internet usage habits, the level of privacy regulation, and the availability of offline social support channels may influence their risk perception and disclosure motivations. Limited evidence regarding outcome variables indicates that in countries with widespread internet access and highly routine platform use, the psychological and social consequences of risky disclosure may show some consistency. These findings provide a theoretical basis for future research to integrate internet usage contexts, social support, and psychological mechanisms, such as identity construction and emotion regulation, in a globalized media environment.

At the platform level, the structural characteristics and interaction logic of social media significantly moderate the expression and outcomes of disclosure behaviors. Platforms with real-name policies, strong interaction chains, and well-developed feedback systems, such as Facebook, may enhance the social rewards of risky disclosure, whereas visually oriented platforms, short-lived content platforms, or platforms centered on familiar social circles may produce more complex effects. This suggests that research and interventions targeting adolescents’ risky online self-disclosure should consider both country-level institutional and usage contexts as well as platform attributes, which may moderate social motivations and risk perceptions (Marciano and Viswanath, 2023; Marciano et al., 2022).

Finally, the moderation analyses underscore the methodological significance of study-level characteristics. Specifically, sample size exerted a substantial effect on effect size variability, whereas individual-level variables such as mean age had limited influence. This finding highlights the need for future studies to employ longitudinal or multilevel designs to capture dynamic interactions between individual and contextual factors. Incorporating cultural-psychological constructs (e.g., collectivism orientation, social desirability sensitivity) and media-structural variables (e.g., visibility, algorithmic recommendation mechanisms) could also enhance model explanatory power (Wang et al., 2023; Metzler and Garcia, 2024; Yuan et al., 2024).

In summary, this study integrated empirical evidence at the national and platform levels to reveal the multilayered mechanisms underlying adolescents’ risky online self-disclosure. Individual psychological motivations serve as the core driving force, while country-level internet usage contexts, digital norms, and social support structures provide institutional and environmental frameworks. Platform characteristics and interaction mechanisms further shape behavioral patterns. These findings not only enrich the theoretical understanding of adolescents’ social media use but also offer important practical insights for digital media literacy education and interventions aimed at reducing risky online behaviors among adolescents.

### Limitations and directions for future research

Although this study made systematic progress in integrating the antecedents, outcomes, and moderating mechanisms of adolescents’ online risky self-disclosure, several limitations remain that warrant further investigation and refinement in future research.

Although this study systematically integrated quantitative research on adolescents’ online risky self-disclosure from 2015 to 2025, the specialized and emerging nature of the topic resulted in the inclusion of only 13 empirical studies. The limited number of studies may affect the stability of overall effect size estimates and reduce the statistical power for testing moderation effects (Borenstein et al., 2021). This issue is particularly evident in subgroup analyses, where some countries or platform categories were represented by only one or two studies, leading to wider confidence intervals for effect size estimates and greater susceptibility of results to individual studies. Consequently, these findings should be interpreted as indicative trends rather than definitive conclusions.

In addition, the limited number of studies reduces the sensitivity of publication bias tests, such as Egger’s test and funnel plot asymmetry assessments, making it difficult to fully rule out the influence of unpublished or small-sample studies on overall conclusions. From a field development perspective, this limitation reflects that research on adolescents’ risky online self-disclosure is still in the early stages of theoretical development and empirical accumulation. Existing studies provide limited coverage of internet usage contexts and digital norms across countries, measurement tools have not yet been fully standardized, study designs are predominantly cross-sectional, and comparative research across different platforms remains scarce.

Future research should expand the volume of empirical evidence and the scope of sample coverage. Studies should strengthen comparisons across countries with different internet usage contexts and digital norms and incorporate longitudinal designs to more comprehensively examine the generality and variability of risky self-disclosure across national, developmental, and platform contexts. Methodological comparability and transparency should also be enhanced, including sharing measurement instruments and coding procedures, and applying more standardized conceptual operationalizations and variable classification systems. By increasing the number of studies, improving research designs, and strengthening methodological rigor, future work is more likely to provide robust evidence on causal mechanisms and advance research on adolescents’ online risk behaviors.

Additionally, this study examined potential moderators, including average age, sample size, country, and platform type, but most moderation effects were not significant, and model heterogeneity remained high. This finding reflects the multidimensional complexity of adolescents’ risky online self-disclosure. Disclosure behaviors are influenced not only by demographic factors but also by the interplay of psychological traits, social relationships, and national internet usage contexts, digital norms, and platform characteristics. However, due to variations in variable operationalization and reporting across primary studies, these potential factors could not be systematically included in the analyses, limiting the explanatory power of the models.

Future research should advance this field by broadening the range of variables and improving data consistency. Given the current limitations in available data and operational definitions, this study was unable to employ more complex statistical approaches, such as multilevel meta-analysis or meta-structural equation modeling (meta-SEM). To address these limitations, future studies should provide more detailed reporting on sample characteristics, platform attributes, and psychosocial variables, which would enable systematic testing of additional potential moderators, including gender composition, socioeconomic status, cultural value orientations, and algorithmic recommendation mechanisms. Building on these methodological improvements, future research could apply multilevel meta-analytic or meta-structural equation modeling approaches to integrate variables across individual, study, and cultural levels from a dynamic systems perspective (Landis, 2013). Such approaches would offer more comprehensive insights into the multifaceted determinants and contextual variability of adolescents’ online risky self-disclosure.

## Data Availability

The original contributions presented in the study are included in the article/Supplementary material, further inquiries can be directed to the corresponding authors.
