# Supplementary material for: Risky online self-disclosure in adolescents: a meta-analytic review of predictors and outcomes
Source: Front Psychol. 2026 Jan 12;16:1734301. doi: 10.3389/fpsyg.2025.1734301 (PMC12833967; doi:10.3389/fpsyg.2025.1734301)
Supplement: Supplementary file 1 [file Data_Sheet_1.pdf]

Appendices

Appendix A: Quality Assessment of Studies Included in the Meta-Analysis

Study	Author (Year)	Sample size	Design	Measurement reliability	Risky self-disclosure measurement	Overall quality
1	Aizenkot (2020)	High	Medium	Medium	Medium	Medium
2	Babilonova et al. (2024)	High	Medium	High	High	High
3	Baumgartner et al. (2015)	Low	Medium	Medium	Medium	Medium
4	Chang et al. (2021)	High	Medium	Medium	High	High
5	Choi et al. (2019)	High	Medium	Medium	Medium	Medium
6	Festl et al. (2019)	Medium	Medium	Medium	High	Medium
7	Hawk et al. (2015)	Medium	Medium	Medium	Medium	Medium
8	Hsieh et al. (2023)	High	Medium	High	Medium	High
9	Koutamanis et al. (2015)	Medium	High	Medium	High	High
10	Paluckaitė & Žardeckait (2019)	Medium	High	Medium	High	High
11	Peluchette et al. (2015)	Medium	Medium	Medium	Medium	Medium
12	Sherman et al. (2016)	Medium	Medium	Medium	High	Medium
13	Yang et al. (2023)	Medium	Medium	Medium	High	Medium

Note. Sample size was rated as *low* (< 200), *medium* (200–1000), or *high* (> 1000). Study design was evaluated based on whether the study employed cross-sectional, longitudinal, or experimental methods and whether appropriate controls were applied. Measurement reliability was assessed according to whether standardized instruments were used and whether reliability coefficients were reported. Risky self-disclosure measurement was rated based on the clarity and specificity with which risky online self-disclosure behaviors were operationalized. Overall quality was determined based on a comprehensive assessment of the four dimensions (sample size, design, measurement reliability, and risky self-disclosure measurement).

Appendix B: Results of Meta-Regression Analyses (Moderators: Mean Age and Sample Size)

Model Type	Moderator	β	p	R^2 (%)	Interpretation
Antecedent（benefit）	Mean Age	-0.12	0.72	-	Not significant
Antecedent（cost）	Mean Age	0.08	0.20	5.70	Limited influence; explained small portion of heterogeneity
Consequence	Mean Age	-0.01	0.47	-	Not significant
Antecedent（benefit）	log(N)	-0.18	<.001	98.73	Significant negative moderation: larger samples show weaker effect
Antecedent（cost）	log(N)	-0.06	0.66	-	Not significant
Consequence	log(N)	-0.01	0.51	-	Not significant

Note. β = regression coefficient; p = significance; R^2 = proportion of heterogeneity explained.

Appendix C: Subgroup Analysis Results: Country and Platform Differences

Model Type	Country	r	95% CI	p	I^2 (%)	Interpretation
Antecedent（benefit）	Netherlands	-	-	-	-	Not analyzed (k < 2)
	Czech Republic	-	-	-	-	Not analyzed (k < 2)
	Lithuania	-	-	-	-	Not analyzed (k < 2)
Antecedent（cost）	Czech Republic	0.47	[0.285, 0.651]	<.001	90	Strong significant association
	Netherlands	-0.06	[–0.329, 0.200]	.63	92	Non-significant relationship
	Lithuania	0.02	[–0.210, 0.257]	.84	96	Non-significant relationship
	China	-	-	-	-	Not analyzed (k < 2)
	Germany	-	-	-	-	Not analyzed (k < 2)
	USA	-	-	-	-	Not analyzed (k < 2)
Consequence	Netherlands	-	-	-	-	Not analyzed (k < 2)
	China	-	-	-	-	Not analyzed (k < 2)
	U.S. & Australia	0.14	[0.083, 0.199]	<.001	0	Consistent cross-cultural effect
	Germany	-	-	-	-	Not analyzed (k < 2)
	Hong Kong	-	-	-	-	Not analyzed (k < 2)
	Israel	-	-	-	-	Not analyzed (k < 2)
	Taiwan	-	-	-	-	Not analyzed (k < 2)
	South Korea	-	-	-	-	Not analyzed (k < 2)

Antecedent (benefit)	General social media	0.39	[–0.51, 1.29]	.40	99.78	Non-significant; high heterogeneity
Antecedent (cost)	General social media	0.11	[–0.09, 0.31]	.26	98.21	Non-significant; high heterogeneity
Consequence	General social media	0.21	[0.12, 0.30]	<.001	99.18	Significant positive relationship
	Facebook	0.14	[0.08, 0.20]	<.001	0	Significant positive relationship
Antecedent (benefit/ cost) / Consequence	Other platforms (WeChat, Weibo, Instagram, SNS, Snapchat)	-	-	-	-	Not analyzed (k < 2)

Note. r = correlation coefficient; CI = confidence interval. Heterogeneity index (I^2) indicates variability between studies.
